# Tuberculous peritonitis in patients on peritoneal dialysis: a 35-year experience from a large medical center in Northern Taiwan

**DOI:** 10.1080/0886022X.2022.2153064

**Published:** 2023-01-12

**Authors:** Tzu-Yi Yang, Ya-Chung Tian, Tzung-Hai Yen, Ming-Yang Chang, Chan-Yu Lin, Shou-Hsuan Liu

**Affiliations:** a Department of Internal Medicine, E-Da Hospital, I-Shou University, Kaohsiung, Taiwan; b Department of Nephrology, Linkou Chang Gung Memorial Hospital, Taoyuan, Taiwan; c Graduate Institute of Clinical Medical Sciences, College of Medicine, Chang Gung University, Taoyuan, Taiwan

**Keywords:** Peritoneal dialysis, tuberculous peritonitis, mortality, catheter removal

## Abstract

**Introduction:**

Tuberculous peritonitis (TBP) is a rare but fatal complication in patients on peritoneal dialysis (PD). In this study, we aimed to determine the demographic features, clinical features, laboratory parameters, and clinical outcomes of PD patients with TBP and to clarify possible risk factors for mortality.

**Materials and methods:**

We retrospectively reviewed 2084 PD patients from January 1985 to December 2019. The diagnosis of TBP was established by positive peritoneal fluid culture for *Mycobacterium tuberculosis*.

**Results:**

18 patients were diagnosed with TBP. The incidence was 2.029 episodes per 1000 patient-years. The most common symptom was fever (94.4%), followed by cloudy effluent (83.3%) and abdominal pain (83.3%). The average peritoneal dialysis effluent (PDE) white blood cell (WBC) count was 172.7 cells/μL. Nine patients (50%) had WBC counts lower than 100 cells/μL and 13 patients (72.2%) had neutrophilic predominant WBC counts. Acid fast stain (AFS) was positive in 7 patients (38.9%). Only 2 patients (11.1%) continued with PD after TB infection, while 10 patients (55.6%) changed to hemodialysis. Seven patients (38.9%) died within 1 year. Significant differences were observed in sex (*p* = 0.040), the presence of diabetes mellitus (*p* = 0.024), and PD catheter removal (*p* < 0.001) between TBP patients with and without mortality. However, none of them was a significant factor for 1-year mortality in multivariate Cox regression model.

**Conclusion:**

Physicians should pay attention to the unusual presentations of peritonitis, especially if symptoms include fever or an initial low PDE WBC count. Catheter removal is not mandatory if early diagnosis and appropriate therapy are available.

## Introduction

According to the 2021 United States Renal Data System (USRDS) annual report, Taiwan had the greatest prevalence (3679 per million population) and the top 2 incidences (529 per million population) of end-stage renal disease (ESRD) in 2019 [1]. Among the population of patients receiving dialysis in Taiwan, peritoneal dialysis (PD) accounted for 8% of the dialysis cases in 2016 [2]. Past studies indicate that patients with chronic kidney disease (CKD) have impaired cellular immunity, which is related to susceptibility to various infections [3–9]. Peritonitis is one of the major complications of PD, and the incidence of PD-related peritonitis was 0.192 ∼ 0.24 episodes per patient-year during 2000 ∼ 2018 in Taiwan [10]. In regard to pathogens, bacterial peritonitis is the most common among PD-related peritonitis, while fungal or mycobacterial peritonitis is less common [11–13].

Tuberculosis (TB) is an ancient disease that is most prevalent in developing regions, such as Southeast Asia, Africa and the western Pacific region [14]. Taiwan used to be one of the countries with a high incidence [15]; nevertheless, there was a 54.2% decrease in the incidence in the period from 2005 to 2021 after the Directly Observed Treatment, Short-course (DOTS) plan was implemented in 2006 [16–18]. Human immunodeficiency virus (HIV), liver cirrhosis, CKD, ethnic factors, rapid immigration, and socioeconomic status are risk factors for TB infection [3,7,19]. A higher incidence was observed in CKD or ESRD patients than in the general population [3,4,7]. Chou et al. reported that the annual TB incidence of the dialysis population (493.4/100000) was 6.9 times higher than that of the general population (71.1/100000) in Taiwan [3].

In this study, we aimed to provide our thirty-five year experience of observing PD patients with TBP at Linkou Chang Gung Memorial Hospital (CGMH), and we aimed to determine the varying demographic features, clinical features, laboratory parameters, and clinical outcomes of PD patients with TBP and to clarify possible risk factors for mortality.

## Materials and methods

This observational cohort study was performed in accordance with the guidelines of the Declaration of Helsinki. Ethics approval (approval number 202201389B0) was obtained from the Institutional Review Board of Chang Gung Medical Foundation in Taiwan without the requirement for a patient consent form because the study was a retrospective review. All the information was anonymized, delinked, and accessible only to the investigator. Finally, all primary data were collected in accordance with the Strengthening the Reporting of Observational Studies in Epidemiology (STROBE) guidelines.

In our retrospective cohort study, we initially reviewed 2084 PD patients at Linkou CGMH over a period of 35 years (from January 1985 to December 2019). Peritonitis was diagnosed when the patient met at least two out of the following three characteristics: (1) consistent clinical features, including abdominal pain or cloudy effluent, (2) peritoneal fluid white blood cell (WBC) count greater than 100/mm^3^ (or 0.1 x 10^9^/L after a dwell time of at least two hours), with > 50% neutrophils, and (3) positive dialysis effluent culture [20]. In terms of TB infection detection, we adopted acid fast stain (AFS) smear, polymerase chain reaction (PCR) and culture for *Mycobacterium tuberculosis* (MTB). The definite diagnosis of TBP in our study was established only by positive peritoneal fluid culture for MTB.

We included baseline demographic features, including sex, age, body mass index (BMI), the cause of ESRD, PD modality (automated PD or continuous ambulatory PD), the duration of PD at enrollment in the study, the presence of diabetes mellitus (DM), and residual urine volume. The clinical features included fever, cloudy effluent, abdominal pain, previous episodes of peritonitis, extraperitoneal TB, and the time from diagnosis to treatment initiation (days). The laboratory parameters included peritoneal dialysis effluent (PDE) features with WBC count, including the percentage of neutrophils and lymphocytes, red blood cell (RBC) count, AFS smear, PCR and culture for MTB, as well as blood analysis with WBC count, hemoglobin (Hb), platelet (PLT) count, C-reactive protein (CRP), blood urea nitrogen (BUN), creatinine, and albumin.

### Statistical analysis

Continuous variables are listed as the mean ± standard deviation (SD). Categorical variables are listed with numbers and percentages. To compare the 2 groups, we used the Mann–Whitney U test to analyze continuous variables and the chi-square test to analyze categorical variables. Univariate Cox regression analysis was used to compare the frequencies of possible risk factors for 1-year mortality. All *p*-values were two-tailed, and a *p*-value < 0.05 was considered statistically significant. All analyses were performed with PASW Statistics for Windows version 18.0 (SPSS Inc.; Chicago, IL, USA).

## Results

**Figure 1.**
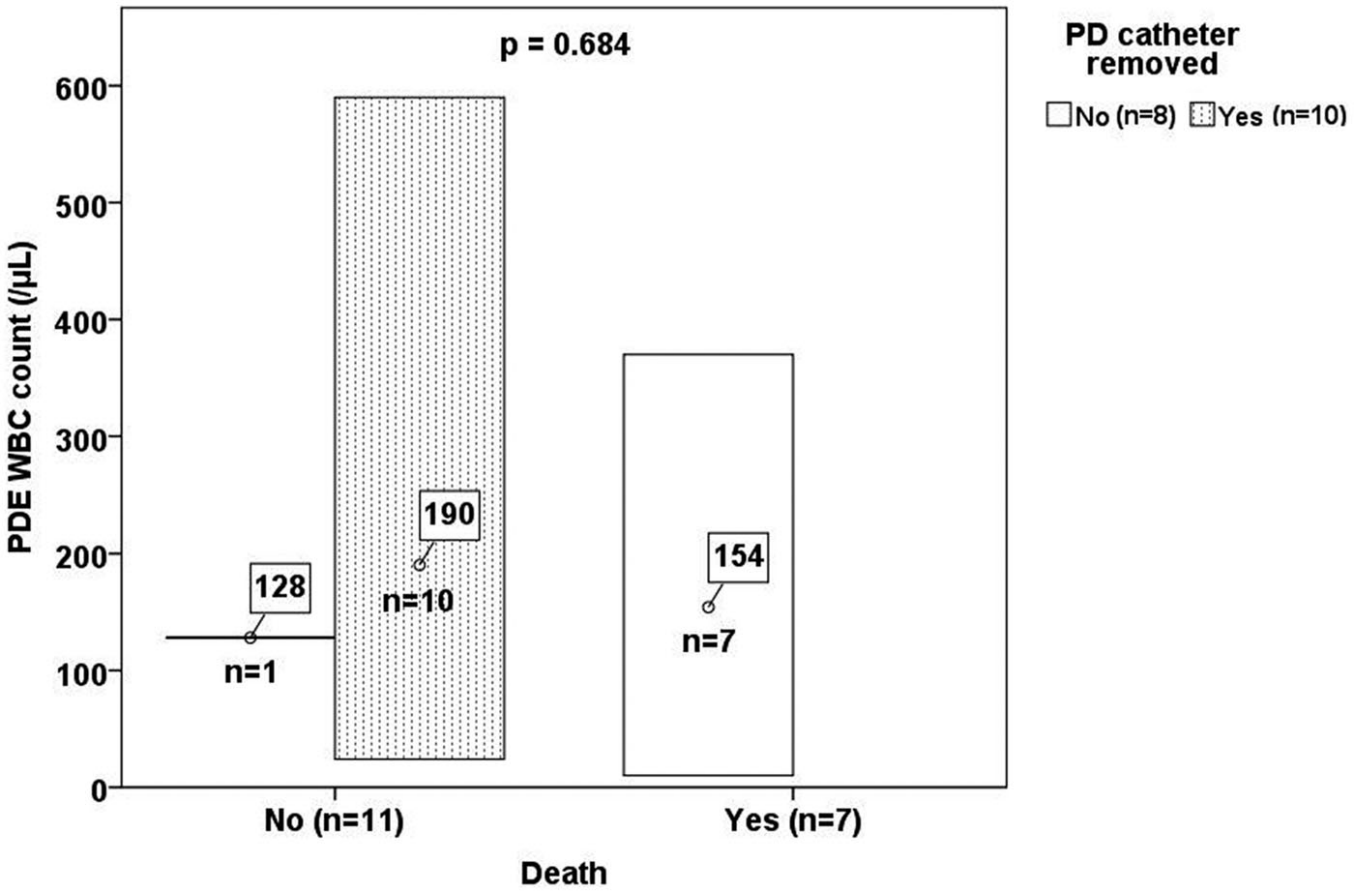
Mean PDE WBC count categorized according to PD catheter removal and mortality.

Of 2084 PD patients, 1026 (49.2%) experienced at least one episode of peritonitis between January 1985 and December 2019. There were 1996 reported episodes of peritonitis, including bacterial (64.13%), fungal (3.36%), tuberculous (0.90%), nontuberculous mycobacterial (0.20%), polymicrobial (7.31%), and culture-negative (24.10%). The cumulative follow-up of all 2084 patients was 106446.9 months. The incidence of all peritonitis was 0.2250 episodes per patient-year, and the incidence of TBP was 2.029 episodes per 1000 patient-years. During the follow-up period, only 18 patients had TBP episodes. The demographic and clinical features are shown in Table 1. The mean age was 56.5 years (range from 19.0 to 87.7 years). The most common symptom in the patients with TBP was fever (94.4%), followed by cloudy effluent (83.3%) and abdominal pain (83.3%). The mean treatment initiation delay was 19.9 days. The PDE and blood laboratory parameters are shown in Table 2. The mean PDE WBC count was 172.7 cells/μL. Thirteen patients (72.2%) had neutrophilic predominant WBC counts. AFS was positive in 7 samples (38.9%). TB PCR was performed in only 5 samples (27.8%), and all were positive (100%). The clinical outcomes are shown in Table 3. Ten patients (55.6%) had to have their catheter removed. The mean duration in days for postperitonitis catheter removal was 21.7 days. 2 patients (11.1%) continued with PD and 10 patients (55.6%) changed to hemodialysis (HD) after TB infection. Seven patients (38.9%) died within 1 year, including 6 who died from sepsis and 1 who died from liver failure secondary to the side effects of anti-tuberculous agents. Figure 1 shows the mean PDE WBC count categorized according to PD catheter removal and mortality. The demographic features, clinical features, laboratory parameters, and clinical outcomes of the PD patients with TBP categorized according to mortality are summarized in Table 4. Significant differences were observed only in sex, the presence of DM, and PD catheter removal. However, the univariate Cox regression analysis demonstrated that only age (hazard ratio = 1.050, *p* = 0.048) was a significant risk factor for 1-year mortality (Table 5). 4 important factors (sex, age, DM, and PD catheter removed) were including into the multivariate model but none of them was a significant factor for 1-year mortality.

**Table 1.** Demographic and clinical features of PD patients with tuberculous peritonitis.

Patient number	Year of diagnosis	Gender	Age (years)	BMI (Kg/m2)	Cause of ESRD	PD vintage (days)	DM	Residual urine volume (ml)	PD modality	Fever	Cloudy effluent	Abdominal pain	Previous episodes of peritonitis	Extraperitoneal TB	Time from diagnosis to treatment initiation (days)
1	1998	Female	60.0	21.5	DM	217	Yes	300	CAPD	Yes	Yes	Yes	1	No	20
2	1999	Female	47.4	23.6	Unknown	1714	No	400	CAPD	Yes	Yes	Yes	0	No	17
3	2001	Male	19.0	23.2	Unknown	751	No	100	CAPD	Yes	Yes	Yes	1	No	13
4	2001	Female	57.5	20.3	DM	434	Yes	500	CAPD	Yes	Yes	Yes	0	No	8
5	2004	Male	44.0	22.5	CGN	912	No	700	CAPD	Yes	Yes	Yes	3	No	24
6	2004	Female	87.7	19.7	CGN	28	No	1000	CAPD	No	Yes	Yes	0	No	47
7	2004	Male	53.4	26.8	CGN	1930	No	0	CAPD	Yes	Yes	Yes	0	No	14
8	2005	Male	50.5	22.2	OU	66	No	800	CAPD	Yes	Yes	Yes	0	No	33
9	2007	Female	48.8	22.9	DM	249	Yes	700	CAPD	Yes	Yes	Yes	0	Yes (miliary)	1
10	2007	Female	63.4	18.1	CGN	2486	No	0	CAPD	Yes	Yes	Yes	4	No	25
11	2008	Female	49.6	20.8	DM	216	Yes	1900	CAPD	Yes	Yes	Yes	0	No	23
12	2011	Female	71.4	20.9	DM	463	Yes	600	CAPD	Yes	Yes	No	0	No	33
13	2012	Male	38.3	22.2	CGN	446	No	150	CAPD	Yes	Yes	Yes	0	Yes (lung)	5
14	2013	Male	82.2	25.1	DM	434	Yes	50	APD	Yes	Yes	Yes	0	No	9
15	2014	Female	32.3	17.8	CGN	1768	No	0	APD	Yes	No	Yes	0	No	14
16	2014	Male	71.7	25.4	CGN	1203	No	200	CAPD	Yes	Yes	No	2	No	36
17	2016	Female	76.9	25.1	DM	627	Yes	0	CAPD	Yes	No	No	0	No	18
18	2018	Male	62.4	18.2	CGN	3166	No	0	CAPD	Yes	No	Yes	1	No	19

BMI: Body mass index; ESRD: end stage renal disease; PD: peritoneal dialysis; TB: tuberculosis; DM: diabetes mellitus; CGN: chronic glomerulonephritis; OU: obstructive uropathy; CAPD: continuous ambulatory peritoneal dialysis; APD: automated peritoneal dialysis.

**Table 2.** Peritoneal dialysis effluent and blood laboratory parameters of PD patients with tuberculous peritonitis.

Patient number	Year of diagnosis	Peritoneal dialysis effluent							Blood						
		WBC count (/μL)	Neutrophil (%)	Lymphocyte (%)	RBC count (/μL)	AFS smear	TB PCR	TB culture	WBC count (/µL)	Hemoglobin (g/dL)	Platelets count (1,000/μL)	CRP (mg/L)	BUN (mg/dL)	Creatinine (mg/dL)	Albumin (g/dL)
1	1998	235	81	19	16	Negative	x	Yes	9600	9.3	167	126.8	74	9.8	3.3
2	1999	190	85	15	12	Negative	x	Yes	8700	8.1	286	x	57	13.4	2.5
3	2001	80	45	10	30	Positive	x	Yes	6300	7.1	353	x	35	11.3	x
4	2001	246	80	20	30	Positive	x	Yes	9600	11.1	291	x	x	6.9	x
5	2004	27	44	53	3	Negative	x	Yes	16700	13	292	117.1	x	13.5	x
6	2004	93	99	1	2	Positive	x	Yes	12600	9.5	452	110.7	42	7.9	x
7	2004	97	95	5	4	Positive	Positive	Yes	14600	8.8	321	222.9	x	10.7	x
8	2005	320	42	58	10	Negative	x	Yes	6100	12.5	292	x	94	10.2	3.3
9	2007	370	78	14	95	Negative	x	Yes	7400	8.1	137	79.3	50	4.2	x
10	2007	262	84	16	17	Positive	x	Yes	8800	9.9	416	122.10	37	6.3	3.3
11	2008	128	86	14	0	Negative	Positive	Yes	12500	8.8	168	53.02	40	5.7	2.8
12	2011	75	89	11	5	Negative	x	Yes	7900	9.4	232	108.92	64.9	3.93	2.01
13	2012	281	99	1	8	Positive	Positive	Yes	9400	7.7	153	59.73	91.1	10.99	3.15
14	2013	590	88	11	25	Positive	Positive	Yes	10000	9.3	443	316.09	69.0	7.22	2.73
15	2014	31	39	61	2	Negative	Positive	Yes	6900	11.5	240	x	56.6	14.22	3.94
16	2014	24	82	18	0	Negative	x	Yes	5900	9	143	63.1	99.5	14.23	3.69
17	2016	50	74	26	0	Negative	x	Yes	12700	10.3	372	11	111.8	5.31	3.36
18	2018	10	30	28	12	Negative	x	Yes	4700	11.4	82	x	100.9	15.97	3.67

WBC: white blood cell; RBC: red blood cell; AFS: acid fast stain; TB: tuberculosis; PCR: polymerase chain reaction; CRP: C-reactive protein; BUN: blood urea nitrogen.

**Table 3.** Clinical outcomes of PD patients with tuberculous peritonitis.

Patient number	Year of diagnosis	PD catheter removed	Catheter removal day number post-peritonitis episode	Keep on PD	Transfer to HD	Death	Cause of death	Survival vintage (days)	Technique survival vintage (days)
1	1998	No	x	No	No	Yes	Sepsis	49	49
2	1999	Yes	92	No	Yes	No	x	x	98
3	2001	Yes	14	No	Yes	No	x	x	14
4	2001	No	x	No	No	Yes	Sepsis	305	305
5	2004	Yes	11	No	Yes	No	x	x	11
6	2004	No	x	No	No	Yes	Sepsis	55	55
7	2004	Yes	30	No	Yes	No	x	x	30
8	2005	Yes	8	No	Yes	No	x	x	8
9	2007	No	x	Yes	No	Yes	Sepsis	109	109
10	2007	Yes	23	No	Yes	No	x	x	23
11	2008	No	x	Yes	No	No	x	x	3997
12	2011	No	x	No	No	Yes	Sepsis	41	41
13	2012	Yes	4	No	Yes	No	x	x	4
14	2013	Yes	3	No	Yes	No	x	x	3
15	2014	Yes	17	No	Yes	No	x	x	17
16	2014	Yes	15	No	Yes	No	x	x	15
17	2016	No	x	No	No	Yes	Liver failure	87	87
18	2018	No	x	No	No	Yes	Sepsis	67	67

PD: peritoneal dialysis; HD: hemodialysis.

**Table 4.** Demographic features, clinical features, peritoneal dialysate effluent laboratory parameters, blood laboratory parameters, peritoneal membrane characteristics, and clinical outcomes of PD patients with TBP categorized according to 1-year mortality.

	Total (*n* = 18)	Survival (*n* = 11)	Death (*n* = 7)	*p* Value
Male	8 (44.4%)	7 (63.6%)	1 (14.3%)	**0.040**
Age (years)	56.5 ± 17.7	50.2 ± 17.8	66.4 ± 13.1	0.063
BMI (kg/m^2^)	22.0 ± 2.6	22.5 ± 2.8	21.2 ± 2.2	0.277
Cause of ESRD				0.128
DM	7 (38.9%)	2 (18.2%)	5 (71.4%)	
CGN	8 (44.4%)	6 (54.5%)	2 (28.6%)	
OU	1 (5.6%)	1 (9.1%)	0 (0%)	
Unknown	2 (11.1%)	2 (18.2%)	0 (0%)	
PD vintage (days)	950.6 ± 904.7	1084.2 ± 795.0	740.6 ± 1087.1	0.239
DM	7 (38.9%)	2 (18.2%)	5 (71.4%)	**0.024**
Residual urine volume (ml)	411.1 ± 492.5	390.9 ± 573.9	442.9 ± 369.0	0.583
PD modality (APD)	2 (11.1%)	2 (18.2%)	0 (0%)	0.231
Fever	17 (94.4%)	11 (100%)	6 (85.7%)	0.197
Cloudy effluent	15 (83.3%)	10 (90.9%)	5 (71.4%)	0.280
Abdominal pain	15 (83.3%)	10 (90.9%)	5 (71.4%)	0.280
Previous episodes of peritonitis	0.7 ± 1.2	0.9 ± 1.4	0.3 ± 0.5	0.516
Extraperitoneal TB	2 (11.1%)	1 (9.1%)	1 (14.3%)	0.732
Time from diagnosis to treatment initiation (days)	19.9 ± 11.8	19.4 ± 9.7	20.9 ± 15.3	0.928
PDE WBC count (/μL)	172.7 ± 153.6	184.6 ± 171.5	154.1 ± 131.1	0.684
PDE Neutrophil (%)	73.3 ± 22.4	71.7 ± 23.7	75.9 ± 21.8	0.786
PDE Lymphocyte (%)	21.2 ± 18.2	23.8 ± 22.1	17.0 ± 9.3	0.856
PDE RBC count (/μL)	15.1 ± 22.3	10.1 ± 10.2	22.9 ± 33.4	0.525
AFS smear	7 (38.9%)	5 (45.5%)	2 (28.6%)	0.474
WBC count (/µL)	9466.7 ± 3250.5	9627.3 ± 3599.7	9214.3 ± 2866.7	0.892
Hemoglobin (g/dL)	9.7 ± 1.6	9.6 ± 1.9	9.9 ± 1.1	0.441
Platelets count (1000/µL)	268.9 ± 111.7	282.5 ± 100.7	247.6 ± 132.6	0.770
CRP (mg/L)	115.9 ± 82.0	136.3 ± 98.8	87.3 ± 46.0	0.570
BUN (mg/dL)	68.2 ± 25.8	64.4 ± 25.4	73.9 ± 27.7	0.346
Creatinine (mg/dL)	9.5 ± 3.8	10.7 ± 3.1	7.7 ± 4.2	0.077
Albumin (g/dL)	3.1 ± 0.5	3.2 ± 0.5	3.1 ± 0.7	0.864
PD catheter removed	10 (55.6%)	10 (90.9%)	0 (0%)	**<0.001**

BMI: Body mass index; ESRD: end stage renal disease; DM: diabetes mellitus; CGN: chronic glomerulonephritis; OU: obstructive uropathy; PD: peritoneal dialysis; APD: automated peritoneal dialysis. TB: tuberculosis; PDE: peritoneal dialysis effluent; WBC: white blood cell; RBC: red blood cell; AFS: acid fast stain; CRP: C-reactive protein; BUN: blood urea nitrogen.

^a^Data are presented as n (%) or mean ± SD.

Bold values denote statistical significance at the *p* < 0.05 level.

**Table 5.** Relative risk of mortality of PD patients with TBP in univariate and multivariate cox regression analysis.

	Univariate (enter method)			Multivariate (enter method)		
	HR	95% CI	*p* Value	HR	95% CI	*p* Value
Male (%)	0.158	0.019–1.321	0.089	4.242	0.091–198.429	0.461
Age (years)	1.050	1.000–1.102	**0.048**	1.078	0.977–1.190	0.136
BMI (kg/m^2^)	0.862	0.649–1.145	0.306			
Cause of ESRD	0.461	0.216–1.084	0.053			
PD vintage (days)	1.000	0.998–1.001	0.417			
Diabetes mellitus (%)	5.174	0.997–15.721	0.050	1.661	0.071–38.850	0.752
Residual urine (ml)	1.000	0.999–1.002	0.821			
PD modality (APD)	25.247	0.002–315979	0.502			
Fever	0.125	0.011–1.382	0.090			
Cloudy effluent	0.425	0.080–2.244	0.313			
Abdominal pain	0.339	0.065–1.772	0.200			
Previous episodes of peritonitis	0.641	0.241–1.703	0.372			
Extraperitoneal TB	1.180	0.141–9.848	0.879			
Time from diagnosis to treatment initiation (days)	1.022	0.956–1.093	0.523			
PDE WBC (/μL)	0.999	0.993–1.004	0.606			
PDE Neutrophil (%)	1.008	0.972–1.046	0.663			
PDE Lymphocyte (%)	0.981	0.933–1.031	0.447			
PDE RBC (/μL)	1.011	0.986–1.037	0.392			
AFS smear	0.523	0.101–2.705	0.439			
WBC count (/µL)	1.000	1.000–1.000	0.798			
Hemoglobin (g/dL)	1.055	0.691–1.612	0.803			
Platelets count (1000/µL)	0.998	0.990–1.005	0.502			
CRP (mg/L)	0.994	0.980–1.008	0.421			
BUN (mg/dL)	1.010	0.979–1.041	0.542			
Creatinine (mg/dL)	0.822	0.644–1.049	0.116			
Albumin (g/dL)	0.560	0.068–4.581	0.589			
PD catheter removed	0.005	0.000–5.590	0.137	0.000	0.000–8.741	0.940

BMI: Body mass index; ESRD: end stage renal disease; PD: peritoneal dialysis; APD: automated peritoneal dialysis. TB: tuberculosis; PDE: peritoneal dialysis effluent; WBC: white blood cell; RBC: red blood cell; AFS: acid fast stain; CRP: C-reactive protein; BUN: blood urea nitrogen.

Bold values denote statistical significance at the *p* < 0.05 level.

A computerized PUBMED review of the English-language literature from January 1990 through June 2022 identified a total of 10 retrospective studies (including 7 single-center and 3 multicenter studies) of PD patients with TBP [4–6,11–13,21–24] (Table 6). The mean follow-up duration ranged from 6 to 25 years. The mean age ranged from 35.4 to 58 years. The mean PD duration ranged from 13.8 to 36 months. The male percentage ranged from 20 to 75%. A total of 10 to 75% of the patients had DM. Symptoms associated with peritonitis, such as fever and abdominal pain, were present in 0 to 100% of the patients, and cloudy PDE was present in 38 to 100% of the patients. The PDE WBC count ranged from 0 to 23040/μL, and the percentage of neutrophils ranged from 57 to 100%. The mean treatment initiation delay ranged from 15 to 77 days, and the treatment duration ranged from 6 to 15 months. A total of 14 to 100% of the patients received catheter removal, and only 1 study mentioned the duration that the postperitonitis catheter was in place (mean 18.7 days). The mortality rate ranged from 0 to 58.3%.

**Table 6.** Summary of published studies reporting PD patients with tuberculous peritonitis.

Year Area	Study Type FU	Patient number	Age (Y)	Vin (M)	Male	DM	AP	Fever	Cloudy PDE	WBC (/μL )	Neu	Treatment initiation delay (day)	Treatment protocol	CR	Mor
1996 HKG [21]	Sin 6 Y	10 of 601 (1.7%)	45.3 (24 ∼ 61)	18.1 (2 ∼ 60)	4 (40%)	1 (10%)	10 (100%)	9 (90%)	7 (70%)	103 ∼ 1530	9 (90%)	77 (14 ∼ 168)	HRZ ± O 9 ∼ 12 M (mean 11 M)	2 (20%) ?	2 (20%)
2000 mixed [11]	Mul 23 Y	52	49.7 (13 ∼ 77)	15.1 (1 ∼ 60)	24 (46%)	?	46 (92%)	39 (78%)	45 (90%)	?	31 (76%)	47	2–4 agents most 6–12 M	27 (53%) ?	13 (25%)
2001 HKG [5]	Sin 6 Y	14 of 790 (1.8%)	58 (29 ∼ 78)	22 (1 ∼ 168)	18 of 38 (47%)	13 of 38 (34%)	14 (100%)	10 (71%)	14 (100%)	125 ∼ 5620	8 (57%)	30 (7 ∼ 57)	HRZO 12 ∼ 15 M	2 (14%) ?	5 (36%)
2004 TWN [6]	Sin 9 Y	5 of 102 (4.9%)	50	27.4	1 (20%)	?	5 (100%)	5 (100%)	?	330 ∼ 23040	3 (60%)	21	?	1 (20%) ?	2 (40%)
2009 mixed [12]	Mul 8 Y	98	51.2 (12 ∼ 76) (*n* = 74)	19 (2 ∼ 84)	39 of 74 (53%)	?	89%	81%	77%	?	65%	48	HRZ ± E or Q	25 of 51 (49%) ?	19 of 56 (34%)
2012 ARE [22]	Sin 12 Y	4 of 89 (4.5%)	52.75 (48 ∼ 57)	17.25 (9 ∼ 48)	3 (75%)	3 (75%)	0 (0%)	0 (0%)	4 (100%)	200 ∼ 350	4 (100%)	?	HRZ 9 M	4 (100%) ?	0 (0%)
2013 IND [4]	Sin 12 Y	11 of 414 (2.6%)	49 (34 ∼ 72)	36 (6 ∼ 108)	8 (73%)	3 (27%)	?	?	?	200 ∼ 6000	9 (82%)	15 (2 ∼ 28)	HRZO	8 (73%) ?	2 (18%)
2014 TWN [23]	Sin 25 Y	4 of 1737 (0.2%)	53.5 (44 ∼ 71)	13.8 (7 ∼ 24)	1 (25%)	3 (75%)	4 (100%)	2 (50%)	4 (100%)	128 ∼ 1290	4 (100%)	?	HRZ ± E at least 9 M	1 (25%) ?	1 (25%)
2016 ZAF [13]	Sin 6 Y	12 of 170 (7.1%)	35.4 (17 ∼ 51)	21.6 (3 ∼ 70)	6 (50%)	2 (17%)	12 (100%)	?	12 (100%)	0 ∼ 3168	9 (75%)	29 (3 ∼ 69)	?	11 (92%) 18.7	7 (58.3%)
2022 mixed [24]	Mul	216	48.9 (*n* = 163)	20.3 (*n* = 143)	51.9% (*n* = 162)	28.3% (*n* = 99)	81.8% (*n* = 137)	67.2% (*n* = 137)	38% (*n* = 137)	mean 1163.4 (*n* = 76)	68.4% (*n* = 85)	43 (*n* = 120)	H 99.1% R 100% Z 81.7% E 45.9% Q 30.6% (*n* = 109)	52.4% (*n* = 167) ?	35.4% (*n* = 206)
2022 TWN Cur	Sin 35 Y	18 of 2084 (0.9%)	56.5 (19 ∼ 88)	31.7 (1 ∼ 106)	8 (44.4%)	7 (38.9%)	15 (83.3%)	17 (94.4%)	15 (83.3%)	10 ∼ 590	13 (72.2%)	20 (1 ∼ 47)	HRZ ± E at least 9 M	10 (55.6%) 21.7	7 (39%)

FU: follow-up duration; Y: year; Vin: PD vintage; M: month; AP: abdominal pain; WBC: initial PDE WBC; Neu: neutrophil predominant; Mor: mortality: CR: catheter removal rate and day number after peritonitis; Tx: treatment; HKG: Hong Kong; Sin: single-center study; H: isoniazid; R: rifampicin; Z: pyrazinamide; O: ofloxacin; Mul: multicenter study; E: ethambutol; Q: quinolone; TWN: Taiwan; ARE: United Arab Emirates; IND: India; ZAF: South Africa; Cur: current study.

## Discussion

To the best of our knowledge, our study is the longest follow-up study about PD patients with TBP in the literature. We provided our single-center experience and performed a literature review of PD patients with TBP.

The diagnosis of TBP is challenging due to its similarities with other diseases, lack of specificity of symptoms, artificial factors of culture, etc [19,25,26].. The symptoms and signs are some of the characteristics that should be taken into account when evaluating TBP. In our study, the most common presentation was fever (17 patients, 94.4%), followed by cloudy effluent (15 patients, 83.3%) and abdominal pain (15 patients, 83.3%). However, these characteristics are similar to and overlap with bacterial peritonitis and fungal peritonitis. AFS, TB PCR and TB culture of the dialysate are the diagnostic tools of TBP. All patients were TB culture positive and could firmly meet the criteria of TBP. However, when using AFS as the only definite diagnostic tool, only 7 patients (38.9%) were positive, which indicates its low sensitivity. The accuracy and the timepoint of the diagnosis affect the subsequent treatment initiation. According to the 2022 International Society for Peritoneal Dialysis (ISPD) peritonitis guideline, culture positivity of the dialysate is the current gold standard, which, nonetheless, may delay the initiation of treatment because of its lengthy process [27]. Several detection tools, such as adenosine deaminase (ADA) in peritoneal dialysate and PCR analysis of MTB, were also used, but the reliability and utility need to be confirmed [27].

It is worth mentioning that there were some confusing situations in predicting TBP by the white cell count and differential in the dialysis effluent. In our patients, the average PDE WBC count was 172.7 ± 153.6 cells/μL. However, 9 out of the 18 patients’ WBC counts were lower than 100 cells/μL, with the lowest count being 10 cells/μL. Therefore, TB PCR should be considered when PD patients initially have clinical features of peritonitis with a low dialysis effluent white cell count.

Although we already know that PD patients are exposed to the risk of TBP in TB-endemic regions, the exact incidence data are limited. In our series, the incidence of TBP was 2.029 episodes per 1000 patient-years with a follow-up period of 35 years. In an earlier study, the incidence of TBP among patients under PD was 5–15 times higher than that in the general population [3–5,19]. In the study by KJ Chou et al. the annual incidence of TB infection among the dialysis population was 4.934 episodes per 1000 patient-years [3]. In a retrospective study by Rapur Ram et al. in India, the incidence was 15.113 episodes per 1000 patient-years with a follow-up period of 12 years (the cumulative follow-up of all 414 patients was 8734 months) [4]. The annual incidence of tuberculous peritonitis (TBP) was 2.954 episodes per 1000 patients within a 6-year period, as reported by Sing Leung Lui et al. in Hong Kong [5]. The incidence discrepancy seems reasonable based on the TB incidence rate among different countries. According to the WHO global TB report [28], TB incidence for the year 2021 is 210 per 100,000 population in India, 57 per 100,000 population in Hong Kong, and 30 per 100,000 population in Taiwan [29].

In previous literature, the mortality of PD patients with TBP has been reported to range from 18 to 58% [4–6,11–13,21,23,24]. One study from the United Arab Emirates reported that all 4 patients with catheter removal survived infection for 3 years [22]. The author stated that catheter removal contributed to a high survival rate. However, data about the duration from placement to removal during a postperitonitis episode and the treatment protocol were lacking. In our study, TB accounted for only 0.88% of all the peritonitis cases. Despite little opportunity for infection, TBP still induced a high mortality. In our study, up to 7 out of the 18 PD patients diagnosed with TBP expired (38.9%). There were significant sex differences in mortality (14.3% versus 85.7%, *p* = 0.04), which indicates that TBP may be more fatal in female PD patients. However, Cox regression analysis demonstrated that sex (hazard ratio = 0.158, *p* = 0.089) was not a significant risk factor for 1-year mortality. The significant sex difference in mortality may be due to age (52.7 versus 59.5 years, *p* = 0.433). Concerning sex, Kang-Ju Chou et al. also reported that the risk of TB infection in CAPD was 11 times higher in females than in males [3]. However, a scoping review of MTB peritonitis in PD patients reported that in a cohort of patients with TB PD peritonitis, the 45 patients who died were less likely to be female (37.8%) [24]. The relationship between sex and mortality in PD patients with TBP is still inconclusive.

Significant differences in mortality were also observed between the patients with or without DM (71.4% versus 28.6%, *p* = 0.024). This is reasonable because DM is related to impaired cellular immunity, which contributes to higher morbidity and mortality of TB infection [11,30,31]. PD has already been shown to be a risk factor for TBP, and our data showed an aggravating inclination that DM is consistently and highly related to mortality of TBP. In our study, Cox regression analysis demonstrated that DM (hazard ratio = 5.714, *p* = 0.050) was almost a significant risk factor for 1-year mortality. Extraperitoneal TB was seen in 2 of the 18 patients, with one who survived and one who expired but with an insignificant outcome (*p* = 0.732). The previous studies did not mention the mortality of TBP of superimposed extraperitoneal TB.

Catheter removal has long been a controversial issue in PD patients with TBP. In our study, 10 patients who underwent catheter removal all transferred to HD, and they all lived. Eight patients did not have their catheters removed, 6 died before continuation, 1 died after continuation, and 1 lived after continuation. It seems that catheter removal after a diagnosis of TBP benefits patient survival. However, Cox regression analysis demonstrated that catheter removal (hazard ratio = 0.005, *p* = 0.137) was not a significant risk factor for 1-year mortality. The discrepancy may be related to female sex (30 versus 87.5%, *p* = 0.015), age (50.2 versus 64.3 years, *p* = 0.094), the presence of DM (10 versus 75%, *p* = 0.005) rather than the mean treatment initiation delay (19.0 versus 21.1 days, *p* = 0.716) between patients with or without catheter removal. In A. Waness’s study, 4 PD patients with TBP received catheter removal intervention, and all patients survived for 3 years [22]. The author concluded that removing the catheter is a better option for treating TBP. In Rapur Ram’s study, there was no significant difference between CAPD removal or maintenance [4]. The more critical point was early diagnosis and appropriate therapy before the decision of catheter removal [4]. A scoping review of MTB peritonitis in PD patients also indicates that catheter removal is not associated with an increased probability of survival [24]. The 2022 ISPD peritonitis guidelines and most studies do not believe that catheter removal is mandatory [4,5,11–13,21,27,32]. However, the day of postperitonitis catheter removal, which is an important parameter that may be related to outcome, was lacking in most previous studies. Future studies can address the effects of delayed catheter removal on mortality.

This study has some limitations. First, this study provides only a single-center experience, and the analysis of multiple center data is needed. Second, two different methods for isolating microorganisms from PDE were used during this study (before and after 1996). The incidence of culture-negative peritonitis (CNP) decreased significantly after changing the culture method in 1996 (35.7 versus 20.7%, *p* < 0.05) in our hospital [33]. The incidence of TBP before 1996 could have been underestimated. In fact, none of the 18 TBP patients was diagnosed before 1996 in our study. Finally, up to 284 of the 481 CNP patients (59%) had not been investigated for TB at the same time for a variety of reasons, such as having clinical improvement after antibiotic treatment (95%) or rapid death. Therefore, there was a relatively high rate (24.10%) of CNP over a period of 35 years in our study. This is another reason for the underestimation of TBP.

In conclusion, the diagnosis of TBP is challenging. The physician should pay more attention to any unusual presentation of peritonitis, especially fever or an initial low PDE WBC count. We suggest that AFS should be performed initially in each patient with peritonitis despite its low sensitivity, and TB PCR should be considered within the initial 5 days if the patient has a poor response to the empiric antibiotics used. Catheter removal is not mandatory if early diagnosis and appropriate therapy are available.
